# Microtubule-Associated Protein 1 Light Chain 3 Interacts with and Contributes to Growth Inhibiting Effect of PML

**DOI:** 10.1371/journal.pone.0113089

**Published:** 2014-11-24

**Authors:** Wei He, Chuan-Xi Hu, Jia-Kai Hou, Li Fan, Yi-Wei Xu, Man-Hua Liu, Shu-Yang Yan, Guo-Qiang Chen, Ying Huang

**Affiliations:** 1 Key Laboratory of Cell Differentiation and Apoptosis of Chinese Ministry of Education, Shanghai Jiao Tong University School of Medicine (SJTU-SM), Shanghai, China; 2 Institute of Health Sciences, Shanghai Institutes for Biological Sciences, University of Chinese Academy of Sciences and Shanghai Jiao Tong University School of Medicine (SJTU-SM), Shanghai, China; German Cancer Research Center, Germany

## Abstract

Previously we reported that the expression of promyelocytic leukemia (PML)-retinoic acid receptor alpha (RARα) fusion gene, which is caused by specific translocation (15;17) in acute promyelocytic leukemia, can enhance constitutive autophagic activity in leukemic and nonleukemic cells, and PML overexpression can sequestrate part of microtubule-associated protein light chain 3 (LC3) protein in PML nuclear bodies, suggesting that LC3 protein also distributes into nuclei although it is currently thought to function primarily in the cytoplasm, the site of autophagosomal formation. However, its potential significance of nucleoplasmic localizations remains greatly elusive. Here we demonstrate that PML interacts with LC3 in a cell type-independent manner as assessed by Co-IP assay and co-localization observation. Overexpressed PML significantly coprecipitates with endogenous and nuclear LC3 protein. Furthermore, a fraction of endogenous PML protein is found to be co-localized with LC3 protein under steady state condition, which is further enhanced by IFNα induction, indicating that PML up-regulation potentiates this interaction. Additionally, DsRed-PML associates with EGFP-LC3 during telophase and G1 phase but not in metaphase and anaphase. Two potential LC3-interacting region (LIR) motifs in PML are required for interaction of PML with LC3 while this association is independent of autophagic activity. Finally, we show that interaction between PML and LC3 contributes to cell growth inhibition function of PML. Considering that PML is an important tumor suppressor, we propose that nuclear portion of LC3 protein may associate with PML to control cell growth for prevention and inhibition of cancer occurrence and development.

## Introduction

Promyelocytic leukemia (PML) gene was discovered as a fusion partner of human retinoic acid receptor alpha (RARα) gene, resulting in PML-RARα fusion protein that is critical for pathogenesis of acute promyelocytic leukemia (APL) [1], [2], [3]. PML protein is characterized by presence of RBCC or tripartite motif (TRIM), which consists of a C3HC4 zinc-finger motif (RING finger), two cysteine-rich and zinc-binding regions (B-boxes), followed by leucine coiled-coil region [4]. Primary and single PML gene transcript undergoes extensive alternative splicing, resulting in expression of seven isoforms designated PML I to PML VIIb. They share the same N-terminal region containing RBCC/TRIM but differ in their C-terminal sequences. Although each PML isoform displays its specific functions, PML proteins generally function as an organizer to PML nuclear bodies (NBs) or PODs (for PML oncogenic domains), which are dynamic and speckled nuclear structures harboring numerous proteins transiently or covalently associated [5]. Therefore, PML and PML NBs are implicated in a wide variety of cellular functions such as transcriptional regulation, protein storage, posttranslational modification, DNA damage response, apoptosis, senescence, angiogenesis, metabolism, antiviral defense and tumor suppression [6], [7], [8], [9]. PML NBs are disrupted and dispersed in microspeckles in the leukemic blasts of APL patients [10], [11], suggesting loss of PML NBs' integrity contributes to leukemogenesis.

Autophagy-related (Atg) 8 protein family is one of highly conserved and critical execution factors during autophagy process that is essential for maintaining cellular homeostasis, controlling quality of proteins and organelles and eliminating pathogens [12], [13], [14]. Multicellular animal Atg8 proteins comprise three subfamilies: microtubule-associated protein 1 light chain 3 (MAP1LC3 or LC3), γ-aminobutyric acid receptor-associated protein (GABARAP) and Golgi-associated ATPase enhancer of 16 kDa (GATE-16) [15]. Among these molecules, LC3B (hereafter referred to LC3) is the first identified mammalian Atg8 protein and regarded as an important marker for assessing autophagic activity so far. During autophagy, cleaved form of LC3 (LC3-I) by Atg4 cysteine proteases is converted into phosphotidylethanolamine (PE) conjugated form (LC3-II), and subsequently LC3-II binds to outer and inner membranes of autophagosomes, thus directly participating in phagophore elongation and autophagosome formation [12]. Recently, accumulating lines of evidence suggest that LC3 acts as a modifier to associate with cargo receptors that sequester cargo into autophagosomes, and promotes selective autophagy through LC3 interacting region (LIR) motif in these receptor proteins [16], [17]. Although LC3 is thought to function primarily in cytosol, the site of autophagosome formation, several lines of evidence indicate that it actually distributes in both cytoplasmic and nucleocytoplasmic areas [18]. However, the function of nuclear pools of LC3 protein have had limited investigation.

Previously we reported that PML-RARα expression significantly enhances constitutively autophagic activity *in vitro* leukemic and nonleukemic cells, and the increased effects of autophagic activity are also found in leukemic cell-infiltrated bone marrow and spleen from *in vivo* leukemic mice [19]. Meanwhile, we unexpectedly found that following overexpression of PML protein, either ectopically or endogenous expressed LC3 is partially co-localized within PML NBs [19]. Here we investigate the interaction of PML with LC3 and its potential functions.

## Materials and Methods

### Cell lines, cell synchronization and reagents

Human prostate cancer cell line PC3, osteosarcoma cell line U2OS and HEK293T cells were purchased from the American Type Culture Collection. Human neuroblastoma cell line SK-N-SH was obtained from cell resource center of Shanghai Institutes for Biological Sciences, Chinese Academy Science, Shanghai, China. Wild-type (WT) and ATG5^−/−^ MEFs were generously provided by Professor Noboru Mizushima [20]. PC3 cells were cultured in Hams' F-12K medium (Gibco, 21127-022) supplemented with 10% fetal bovine serum (FBS, Gibco, 26140). SK-N-SH, HEK293T, U2OS, and WT or ATG5^−/−^ MEFs were cultured in Dulbecco's modified Eagle's medium (DMEM, HyClone, SH30022.01B) containing 1% penicillin and 1% streptomycin, supplemented with 10% FBS. All cell lines were incubated in 5% CO_2_/95% air humidified atmosphere at 37°C. Metaphase synchronization was achieved by treatment with 200 ng/ml nocodazole in complete media for 18 hours. Recombinant human IFN-α 2A was purchased from Peprotech (300-02AA) and doxorubicin was obtained from Sigma (44583-10MG). EBSS was made according to the media formulations as described previously [21].

### Plasmids and transfection

pEGFP-LC3B plasmid was constructed by our group [19], and pFlag-CMV4-PML I was a generous gift from Dr. Jian-Hua Tong in Shanghai Institute of Hematology (SIH). pFlag-CMV4-PML IV was constructed by PCR strategy from pFlag-CMV4-PML I into pFlag-CMV4 expressing vectors. DsRed-PML IV and pLVX-Flag-PML IV plasmids were respectively made by a swap of PML IV cDNA into DsRed or pLVX vectors (Clontech, 632164) from pFlag-CMV4-PML IV. The sequences of cDNA inserts were confirmed by sequencing. The Flag tagged PML mutant1, mutant2 and double mutant were generated by PCR methods with site mutation sequence and then cloned into pCMV4 vector and pLVX vector. Transient transfection was performed with HilyMax Transfection Reagent according to the manufacturer's procedures (Dojindo Molecular Technologies, H357).

### shRNA design and viral infection

Complementary oligonucleotides against PML (siPML#1 and siPML#2), referred to the sequences of PML si1 and PML si3 in the reference [22], were synthesized, annealed and ligated into pSIREN-RetroQ vector according to Knockout RNAi Systems User Manual (Clontech Laboratories, Inc. Mountain View, CA). These siRNA and non-specific siRNA (NC) plasmids with pSIREN-RetroQ or pLVX vector were co-transfected with packaging plasmids including pCMV-Gag pol, VSV-G or pMD2G and PSPA2 into HEK293T cells to produce retrovirus or lentivirus. Forty-eight hours later, the viral supernatants were collected, filtered through 0.45 µm membrane (Millipore) and respectively added into 293T cells incubated with the medium containing 1 µg/ml polybrene (santa cruz, sc-134220). Stably expressed cells were selected by 1 µg/ml puromycin after viral infection for 48 hours.

### Protein extract, subcellular fractionation and immunoprecipitation

About 3×10^7^ of transfected HEK293T cells were harvested and cytosol and nuclei fractions were isolated as previously described [23]. 1×10^7^ cells were harvested with immunoprecipitation lysis buffer (50 mM Tris-HCl, pH 7.4, 150 mM NaCl, 1 mM EDTA, 1% NP-40, 1 mM PMSF, protease inhibitor cocktail). After brief sonication, the cell lysates were centrifuged with 12000 rpm at 4°C for 10 minutes. The lysates were pre-cleaned with normal IgG at 4°C for 2 hours and supernatants were incubated with mouse anti-Flag M2 Affinity Gel (Sigma, A2220) overnight at 4°C. After immunoprecipitation, the beads were washed with washing buffer (PBS plus 0.05% Tween) for five times and the precipitates were analyzed by western blot with indicated antibodies.

### Western blots

The whole cell lysates were extracted in PBS, plus 2×SDS, equally loaded onto SDS-PAGE, and subsequently transferred to the Immobilon PVDF Transfer Membranes (Millipore Corporation Billerica, MA). After blocking in 5% non-fat milk at room temperature for 1 hour, the membranes were incubated with the indicated primary antibodies overnight at 4°C, followed by HRP-linked secondary antibodies (Cell Signaling, 7074). The signals were detected by chemiluminescence phototope-HRP kit (Millipore, WBKLS0500) according to the manufacturer's instructions.

### Antibodies

Anti-PML antibodies used in immunofluorescence assay and western blot were respectively purchased from Santa Cruz (mouse PML clone PG-M3) and Bethyl (rabbit A301-167A). Anti-LC3 antibody was obtained from Sigma Aldrich (L-7543). Anti-α-tubulin-HRP-DirecT antibody (PM054-7) and anti-DDDDK-tag (mouse, M185-3L) for testing the expression of Flag tagged proteins were purchased from Medical and Biological Laboratories. Anti-myc-tag antibody was got from Cell Signaling (rabbit, 2272). Lamin B (sc-6216), Daxx (sc-7152) and Sp100 (sc-16328) were obtained from Santa Cruz. β-Actin was purchased from Calbiochem (CP01).

### Confocal microscopy and immunofluorescence

U2OS and other indicated cell lines were seeded on glass coverslips and then transfected with the indicated plasmids using HilyMax Reagent. After transfection for 24 hours, the cells were fixed in 4% paraformaldehyde PBS at room temperature for 30 minutes. The fixed cells were washed in PBS and permeabilized with methyl alcohol for 20 minutes. After washing with PBS for 3 times, the cells were blocked with 1% BSA in PBS at room temperature for 2 hours. Then, the cells were incubated with indicated primary antibodies overnight at 4°C and washed for 3 times on the following day. Finally the cells were stained with corresponding secondary antibodies (Santa Cruz), followed by washing and finally the coverslips were mounted on glass slides. Fluorescent signaling was visualized under NICON fluorescent microscope. PML nuclear spots were detected and visualized by anti-PML or Flag antibody under confocal microscopy. Colocalization of PML with LC3 was quantified in the section where more and clear PML nuclear bodies could be observed. Images show a single z-section. For calculating percentages of colocalization, numbers of PML nuclear body colocalized partially or completely with Myc-LC3 or LC3 per cell were counted, and then colocalization percentage of PML and LC3 per cell was calculated based on numbers of PML NBs colocalized with LC3 and total numbers of PML NBs. For each experiment, 30–50 cells were observed. Data of colocalization percentage show mean percentage with S.D by analyzing 30 or 50 cells in an independent experiment.

### Colony Formation Assay

One hundred HEK293T cells transfected with indicated plasmids were seeded on 6-well plates. The cells were cultured *in vitro* for 15 days and stained with 1% crystal violet after fixation with methyl alcohol. Visible colonies were counted.

### CCK-8 assay

Plates were pre-incubated in 5% CO_2_/95% air humidified atmosphere at 37°C and followed by seeding 500 HEK293T cells into per well of 96-well plates. After the cells were cultured for the indicated days or treated with various concentrations of doxorubicin for 24 hours, 10 µl CCK-8 solutions (CK04, Dojindo Molecular Laboratories) were added into each well and incubated at 37°C for 2 hours. The absorbance was measured by a Synergy H4 Hybrid Reader (BioTek) at a wavelength of 450 nm. Cell growth or cytotoxic activity induced by doxorubicin was assessed by cell numbers that had been calculated based on the standard curve between absorbance values and cell numbers. Each sample was triplicate.

### Statistical analysis

Student's t-test was used to evaluate the differences between two groups. A p value of less than 0.05 was considered statistically significant.

## Results

### PML interacts with overexpressed and endogenous LC3 proteins

**Figure 1:**
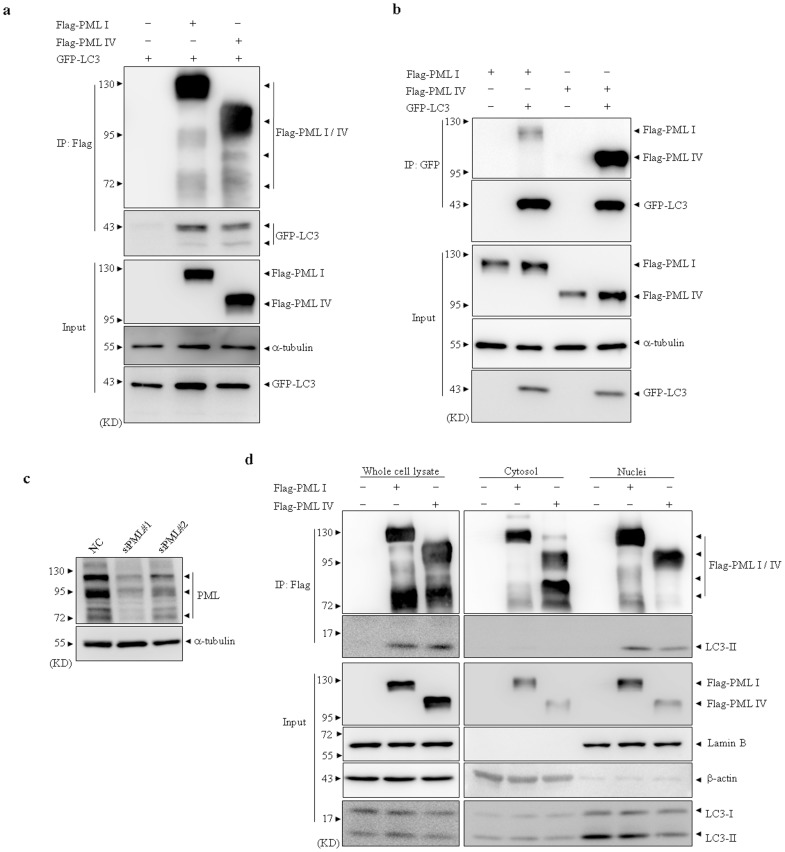
PML interacts with overexpressed and endogenous LC3 proteins. (a–b) HEK293T cells were transiently transfected with the indicated plasmids. After transfection for 48 hours, whole cell lysates were harvested and Co-IP assay was performed by Flag (a) or GFP (b) antibody. Then the indicated proteins were detected by western blot. (c) HEK293T cells were stably transfected with siPML#1, siPML#2 or NC. The expression of PML protein level was detected by PML antibody with α-tubulin as loading control. (d) siPML#1-expressing HEK293T cells were transiently transfected with Flag tagged shRNA-resistant PML I, PML IV or empty vectors, then fractionated cytosol and nuclei together with whole cell lysates were applied to IP by Flag antibody. Endogenous LC3 protein was detected in immunoprecipitate by western blot. 10% cell lysates (input) was used as a positive control. All experiments were repeated for three times and similar results were obtained.

To test whether PML interacts with LC3, human embryonic kidney cell line HEK293T was transiently co-transfected with GFP-LC3 and Flag tagged PML I or PML IV expressing plasmids, or empty vector as a negative control. After transfection for 48 hours, co-immunoprecipitation (Co-IP) assay was performed. As shown in Figure 1a, Flag tagged PML I/IV and GFP-LC3 proteins were found in the whole-cell lysates, indicating effective transfection. Anti-Flag antibody could efficiently precipitate Flag-PML I/IV proteins, suggesting an effective and specific immunoprecipitation by Flag antibody. As we expected, GFP-LC3 proteins were detected in Flag antibody-pulled down immunoprecipitates of PML I/IV but not that of vector expressing (Figure 1a), suggesting that overexpressed PML could interact with GFP-LC3. Vice versa, Flag-PML I and IV proteins could also significantly be coprecipitated with GFP-LC3 by anti-GFP antibody albeit with different pull down effects (Figure 1b).

To confirm above-observed interaction of PML with LC3, we generated HEK293T cells stably expressing two pairs of small interfering RNA (siRNA) specifically against PML (designated siPML#1 and siPML#2 respectively) (Figure 1c). siPML#1 expressing cells, demonstrating remarkably silenced PML expression resulting in clear background to exclude possible disturbance of expression of distinct endogenous PML isoforms, were further transiently transfected with Flag-tagged and siRNA-resistant PML I, PML IV or empty vector. After transfection for forty-eight hours, cytosol and nuclei of transfected HEK293T cells were respectively fractionated, as verified by corresponding subcellular resident proteins including cytosolic protein β-actin and nuclear protein lamin B. Further, these protein extracts together with whole cell lysates (WCL) were applied for immunoprecipitation (IP) assay. The results showed that anti-Flag antibody could effectively pull down Flag-PML I/IV proteins from WCL, cytosol and nuclei fractions, together with endogenous and particularly LC3-II proteins derived from WCL and nuclei but not cytosol (Figure 1d), suggesting PML interacted with endogenous and nuclear LC3 proteins.

### Transfected and induced expression of PML increase part of LC3 proteins to co-localize with PML NBs

**Figure 2:**
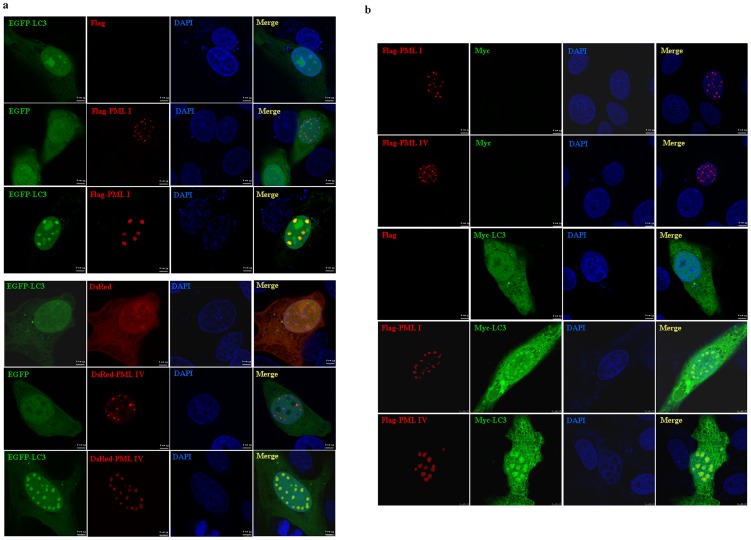
Effects of transfected expression of PML on distribution of LC3 protein. (a–b) U2OS cells were transiently co-transfected with two pairs of expressing plasmids, EGFP-LC3 and Flag-PML I (or DsRed-PML IV) (a) or Flag-PML I/IV and Myc-LC3 (b) together with their corresponding empty vectors. After transfection for 24 hours, the cells were fixed and observed by confocal microscopy. Representative colocalization images of overexpressed PML I/IV and LC3 were shown (scale bar  = 7.5 µM).

Human osteosarcoma cell line U2OS was transiently co-transfected with GFP-LC3 and Flag-PML I, DsRed-PML IV or their corresponding empty plasmids. In line with the previous reports [24], PML I and PML IV proteins demonstrated dispersed and punctuated nuclear structures. Consistent with our previous findings [19], ectopic expression of PML I and IV significantly sequestered a fraction of LC3 protein within PML NBs leading to complete co-localization of LC3 with PML, while this phenomenon could not be seen in DsRed, Flag or EGFP plasmid-transfected cells (Figure 2a), suggesting specific events for PML expression and no artefactual EGFP signal from bleed-through of DsRed-PML IV. Similar co-localization effect was also found in U2OS cells (Figure 2b) when co-transfected with Flag-PML I/IV and Myc-LC3 expressing plasmids, thus excluding the possibility of aberrant localization with GFP tag. Subsequently, we found the effects of PML-induced LC3 recruitment within PML NBs occurred in several human cell lines tested, including human cervical cancer cell line HeLa, laryngeal carcinoma cell line Hep2, prostate cancer cell line PC3, HEK293T and neuroblastoma cells SK-N-SH. These results suggested that PML promoting recruitment of partial LC3 protein in PML NBs is in a cell type-independent manner.

**Figure 3:**
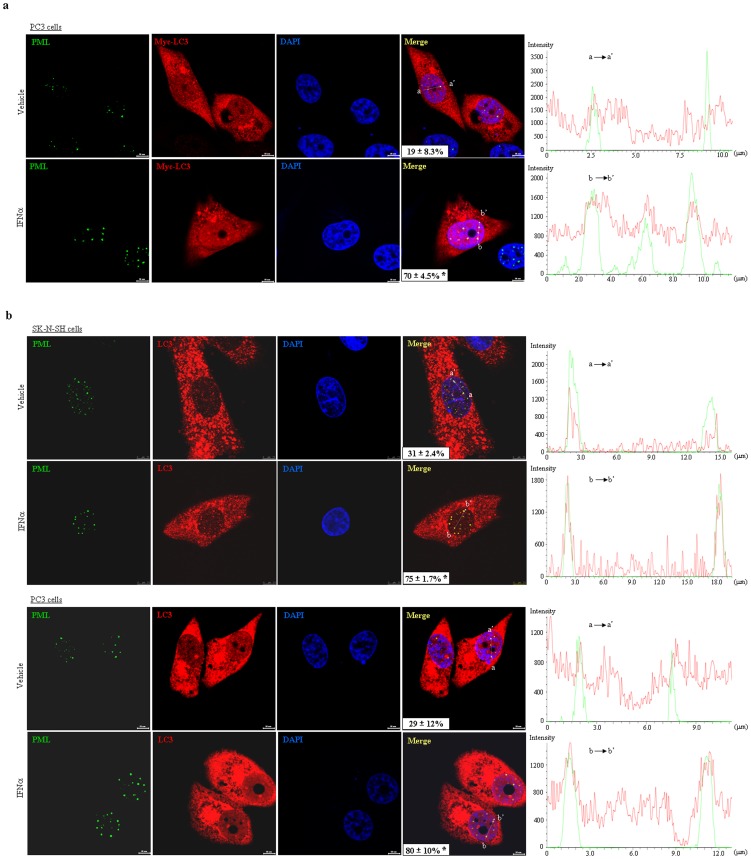
Effects of IFNα-induced expression of PML on localization of LC3. (a–b) PC3 cells were transiently transfected with Myc-LC3 and followed by treatment of IFNα at 2000 IU/ml for 48 hours (a), while SK-N-SH and PC3 cells were respectively treated with IFNα (2000 IU/ml for SK-N-SH and 8000 IU/ml for PC3) or vehicle for 48 hours (b). Localization of PML and LC3 proteins were detected by PML and Myc (a) or LC3 (b) antibodies. The representative images of treated cells as indicated were shown [scale bar  = 10 µM in (a) and (b) for PC3 cells); scale bar  = 7.5 µM in (b) for SK-N-SH cells]. PML NBs-colocalized with Myc-LC3 or LC3 were quantified with line scan analysis (right graph) by observing overlap of fluorescence intensity peaks along profiles spanning PML NBs (a→a' or b→b') as indicated in merge image. Values (x±SD) in merged images represented mean percentages of PML NBs colocalized with LC3 per cell (with S.D) by observing 50 PC3 or SK-N-SH cells in an independent experiment. The symbol * indicated p<0.01 compared to the cells treated with vehicle. All experiments were repeated for three times and similar results were obtained.

To exclude possibly artificial effect of PML overexpression on LC3 localization, PC3 cells were transiently transfected with Myc-LC3 and followed by treatment with and without interferon (IFN) α for 48 hours in that IFNα can specifically upregulate PML gene in a variety of cells [25]. Indirect immunofluorescence assay showed that compared to the cells treated with vehicle, IFNα significantly increased numbers and intensities of PML NBs in the cells (Figure 3a–b), which was consistent with previous reports [25], [26]. As shown in Figure 3a, Myc-LC3 had a diffuse distribution pattern, which localized in cytoplasm with a relatively large amount accompanied with some aggregates and less amount in nucleocytoplasm together with a few smaller foci at steady state, whereas IFNα could induce clearly and large nuclear Myc-LC3 foci that significantly colocalized with PML NBs. Colocalization percentage of PML NBs with Myc-LC3 in vehicle and IFNα-stimulated cells was quantified with line scan analysis by fluorescence intensities overlap along profiles spanning PML NBs per cell. In control cells, there was part of PML NBs colocalized with Myc-LC3, while IFNα treatment could significantly increase the colocalization percentage of PML and Myc-LC3 (19±8.3% for control; 70±4.5% for IFNα), as analyzed by 50 Myc-LC3 expressing cells. Further, we tested whether this co-localization effect could happen in endogenous PML and LC3 proteins. As expected with the notion that LC3 exerts function primarily in cytosol, endogenous LC3 proteins were distributed mainly in cytoplasma and less in nucleocytoplasmic regions in both PC3 and SK-N-SH cells, which is consistent with the findings that LC3 shuttles between these two compartments [18]. Similar to the observed effect of overexpressed Myc-LC3 and endogenous PML proteins, by calculating percentages of PML bodies colocalized with LC3 in 50 SK-N-SH cells (upper parts, Figure 3b), 31% PML NBs demonstrated to colocalize with LC3 under steady state condition and IFNα further significantly enhanced the colocalization effect of PML NBs with LC3, depicting that 75% PML NBs were colocalized with LC3. Similar results could also be seen in PC3 cells (lower parts, Figure 3b), as assessed by line scan analysis and calculating percentage of PML NBs colocalized with LC3 (29±12% for vehicle; 80±10% for IFNα). These results strongly indicated PML could interact with LC3 under physiological condition and this effect can be intensified by PML expression.

**Figure 4:**
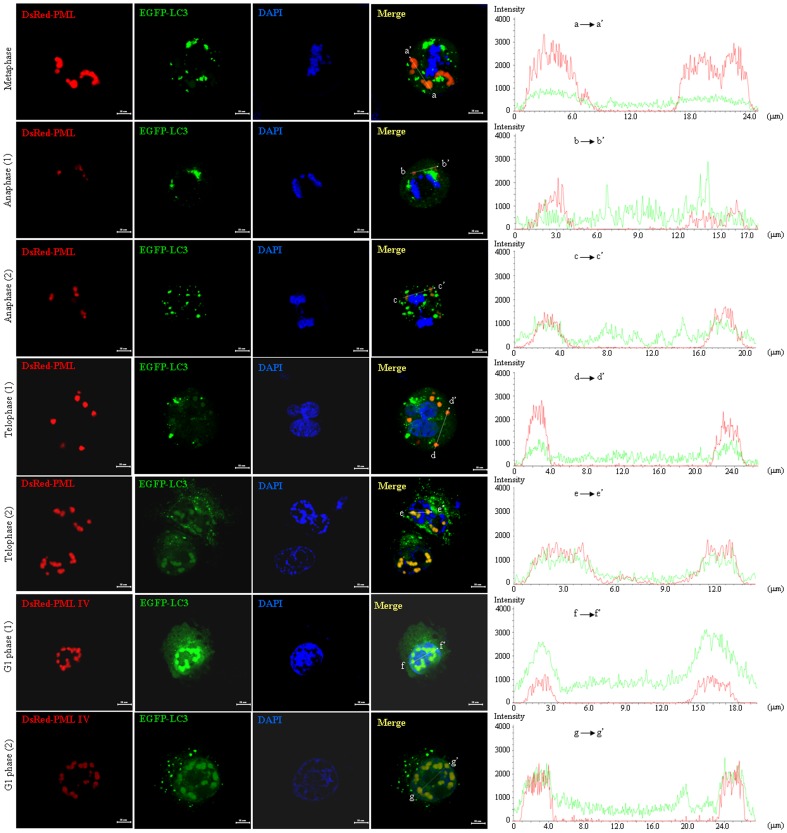
Colocalization effect of overexpressed PML and LC3 during cell cycle. PC3 cells were transiently transfected with DsRed-PML IV and GFP-LC3 expressing plasmids and followed by treatment with nocodazole at 200 ng/ml for 18 hours. The treated cells were harvested by mitotic shake off, seeded onto coverslips and released from nocodazole block for 1 and 2 hours. Then the cells were fixed after staining with hoechest 33342 (blue) for observation with confocal microscopy. Representative images of dividing and G1 phase cells were observed (scale bar  = 10 µM). Right graph presented line scan analysis for colocalization of DsRed-PML and GFP-LC3 based on fluorescence intensity profiles across PML NBs as indicated on left merged images. Experiments were repeated for three times and similar results were obtained.

Recently, Palibrk et al have reported that PML bodies form stable interacting complex with early endosomes after entry into mitosis and these two compartments stably associated throughout mitosis and dissociate in the cytoplasm of newly divided daughter cells [27]. To address whether PML interacted with LC3 protein dependent on a specific cell cycle stage, PC3 cells were co-transfected with DsRed-PML IV and GFP-LC3 expressing plasmids. After transfection for 24 hours, the cells were treated with 200 ng/ml nocodazole for 18 hours to arrest the cells in metaphase and then released from nocodazole block for 1 and 2 hours. After release for 2 hours, majority cells entered into G1 phase. Then, transfected cells in different phases of cell cycle were randomly observed by confocal microscopy. As shown in Figure 4, GFP-LC3 partially colocalized in cytoplasmic DsRed-PML protein during early phase of telophase (telophase 1) and completely colocalized within nuclear DsRed-PML NBs during later stage of telophase (telophase 2) and G1 phase while this phenomenon could not be seen in metaphase and anaphase, as assessed by line scan analysis. These results suggested that PML associates with LC3 may occur in telophase and G1 phase.

### Interaction between PML and LC3 proteins is independent of autophagy

**Figure 5:**
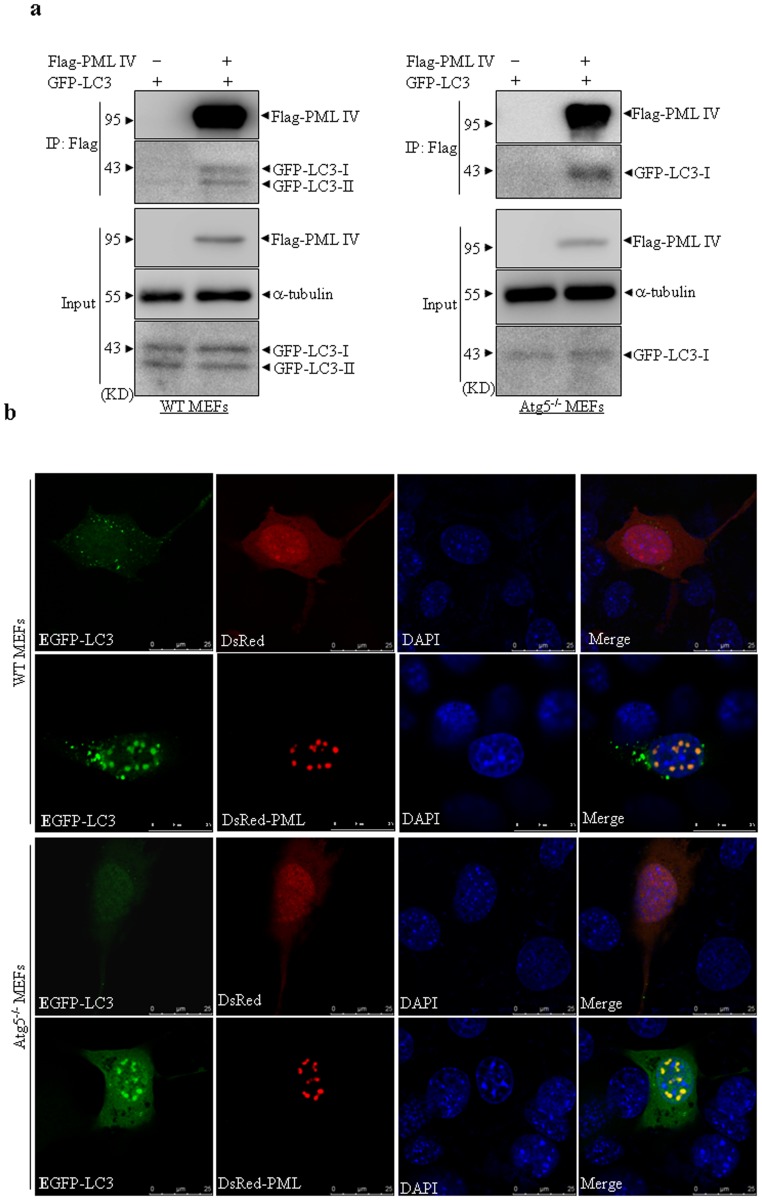
Interaction of PML and LC3 is independent of autophagic activity. (a) Wild type (WT) and Atg5^−/−^ MEF cells were respectively transfected with the indicated plasmids. After transfection for 48 hours, whole cell lysates were harvested and immunoprecipitated with Flag antibody, followed by immunoblots with GFP antibody. 10% cell lysates (input) was used as a positive control. (b) Indicated cells were co-transfected with EGFP-LC3 and DsRed-PML IV or DsRed vector, respectively. Following transfection for 24 hours, the cells were fixed and observed under confocal microscopy. Representative colocalization images of EGFP-LC3 and DsRed-PML in indicated cells were shown (scale bar  = 25 µm). All experiments were repeated for three times and similar results were seen.

Autophagy process is regulated by a series of autophagy-related genes such as Beclin1, mTOR, LC3 and Atg5 that control distinct stage of autophagy (initiation, progression and maturation) [28], [29]. To determine whether PML-interacting with LC3 was dependent on autophagy, wild-type (WT) and Atg5 knockout (Atg5^−/−^) mouse embryonic fibroblast cells (MEFs) were transiently co-transfected with GFP-LC3 and Flag-PML IV or empty vector, respectively. Considering PML IV has been one of the most studied isoforms of PML recently, which has several functions such as growth inhibition, apoptosis, senescence, destabilization of c-Myc and antiviral response [30], [31], we used PML IV isoform plasmid as a studying tool in the following experiments. Consistent with previous report [20], conversion of LC3-I into LC3-II was found in WT MEFs but not in Atg5^−/−^ MEFs when these cells were treated with nutrient-free EBSS, the medium of autophpagy inducer, for 30 minutes, suggesting that autophagosome formation was impaired and autophagic activity was compromised in Atg5^−/−^ MEFs. As shown in Figure 5a, Flag-PML IV could coprecipitate with GFP-LC3 regardless the presence and absence of Atg5 gene expression. Noticeably, overexpressed GFP-LC3 protein processing from GFP-LC3-I to GFP-LC3-II could be easily found in cell lysate (input) of WT MEF cells, which is not seen in 293T cells (Figure 1a and 1b). This was possibly due to different constitutive autophagy activity or different transfection potency existed in these two cell lines. Under latter situation, there might not be enough time for GFP-LC3 processing when producing more GFP-LC3-I proteins such as in 293T cells, since we found that transfection efficiency was much higher in 293T than in MEF cells. Meanwhile, we also found that overexpressed PML IV recruited partial GFP-LC3 proteins within PML NBs either in WT or Atg5^−/−^ MEFs (Figure 5b). These data proposed that PML interacts with LC3 proteins independent of autophagic activity.

### LIR motifs are required for interaction of PML with LC3

**Figure 6:**
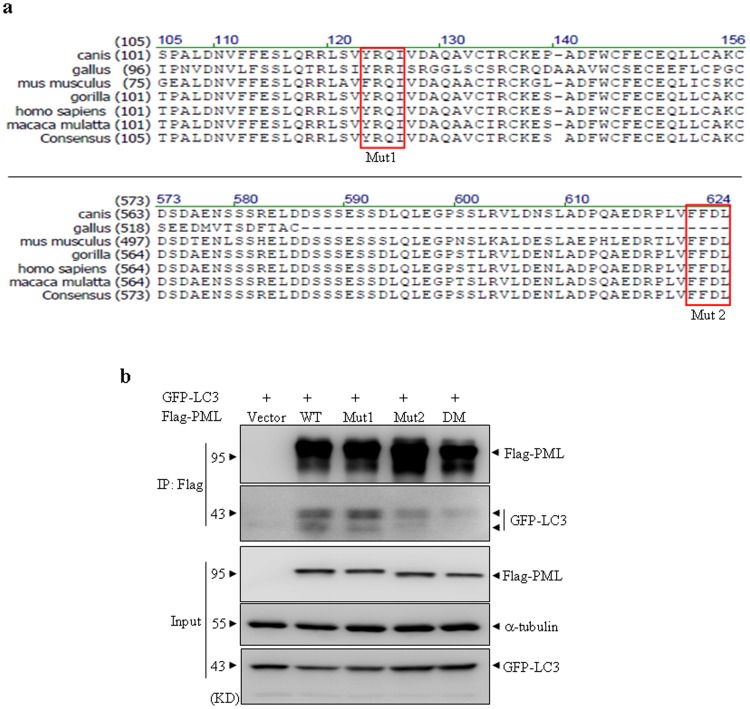
LIR motifs are required for interaction of PML and LC3. (a) Alignments of a portion of PML protein sequence across several mammalian species showed two conserved LIR motifs as indicated in the rectangles. Two potential LIR motifs were respectively mutated into the mutant form AXXA, designated mutant 1 (Mut1) and mutant 2 (Mut2). (b) HEK293T cells were transiently transfected with the indicated plasmids and Co-IP was performed with Flag antibody. The indicated proteins were analyzed by immunoblotting. 10% cell lysates (input) was used as a positive control. DM represents double mutant PML plasmid with the mutated sites of both mutant 1 and mutant 2. These experiments were repeated for three times and similar results were obtained.

Accumulating evidence indicates that LC3-interacting region (LIR), W/Y/F-x-x-L/I/V (x means any amino acid), mediates a specific interaction between LC3/GABARAP family proteins and selective autophagic substrates and cargo receptors such as p62 and NBR1 [16]. This specific region localizing within these substrates and receptors can bind to two hydrophobic pockets conserved in LC3 family proteins. A series of studies show that mutation of one or two conserved critical W/Y/F and/or L/I/V residues into alanine in this motif results in remarkable decrease of interaction with LC3 protein [32], [33], [34], [35]. With this notion in mind, we blasted amino acid sequences of PML from a series of mammalian species including gorilla, mus musculus, gallus and human beings et al. As expected, there are two potential conserved LIR sites (Y/FRQI and FFDL) existed in PML protein, which are localized in amino acid (aa) 124–127 and aa 621–624, respectively (Figure 6a). To test whether these LIR motifs contributed to interaction of PML and LC3, several mutated PML expressing plasmids were generated from wild type PML IV plasmid, in which tyrosine (aa 124) and isoleucine (aa 127) residues in Y/FRQI were mutated into alanine (ARQA for Mutant 1), Phenylalanine (aa 621) and Leucine (aa 624) residues in FFDL were mutated into alanine (AFDA for Mutant 2), together with combination of Mutant 1 and Mutant 2 (ARQA^124–127^/AFDA^621–624^ for Double mutant, designated DM). Then HEK293T cells were transiently co-transfected with GFP-LC3 and Flag-tagged WT or individual mutant PML plasmids. As shown in Figure 6b, anti-Flag antibody could effectively precipitate all Flag tagged WT and mutant PML proteins, indicating similar pull down efficiency of CO-IP. Mutant 2 but not WT and Mutant 1 PML lost its interaction capability with LC3 protein to a degree, while DM PML failed to co-precipitate LC3 protein. These data suggested that two LIR motifs, particularly the second one, are required for PML interacting with LC3.

**Figure 7:**
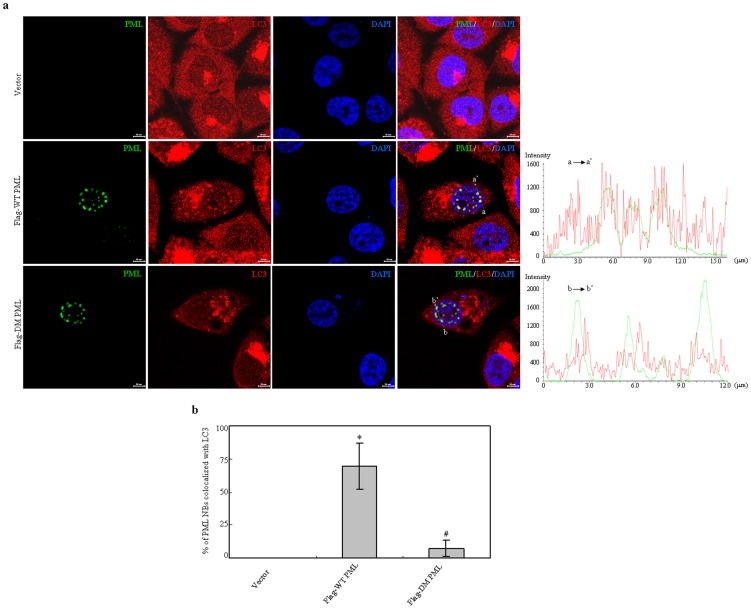
Effects of wild type and double mutant PML on localization of endogenous LC3 protein. PC3 cells were transfected with Flag tagged WT and double mutant (DM) PML expressing plasmids. After transfection for 48 hours, the localization of PML and LC3 proteins were analyzed with Flag and LC3 antibodies. (a) Representative images were captured by confocal microscope (scale bars  = 10 µM). Line scan analysis right was applied to quantify colocalization of LC3 and Flag tagged WT PML or DM PML crossing PML NBs as indicated on left merged images. (b) Quantification of percentages of PML NBs colocalized with LC3 per cell in part (a) was presented. Data presents mean percentage with bar as S.D by analyzing 30 cells in an independent experiment. The symbols * and # indicate p<0.01 compared with the cells expressing empty or Flag-WT PML plasmids, respectively. All experiments were repeated for three times and similar results were obtained.

To further verify this effect, we observed the localization of endogenous LC3 proteins in PC3 cells transfected with Flag tagged WT or DM PML expressing plasmids, together with pLVX vector as control. DM PML demonstrated similar nuclear distribution pattern compared with WT PML. Similar to the effects of IFNα induction (Figure 3), high resolution images revealed that overexpression of WT PML but not DM PML could significantly enhance the recruitment of endogenous LC3 protein in PML NBs, as analyzed by line scan and calculating percentages of PML NBs colocalized with LC3 in 30 cells for each group (73±15% for WT PML, 9±6% for DM PML) (Figure 7a–b). These results suggested two LIR motifs in PML indeed contribute to interaction of PML with LC3.

### Interaction between PML and LC3 contributes to cell growth inhibiting function of PML

**Figure 8:**
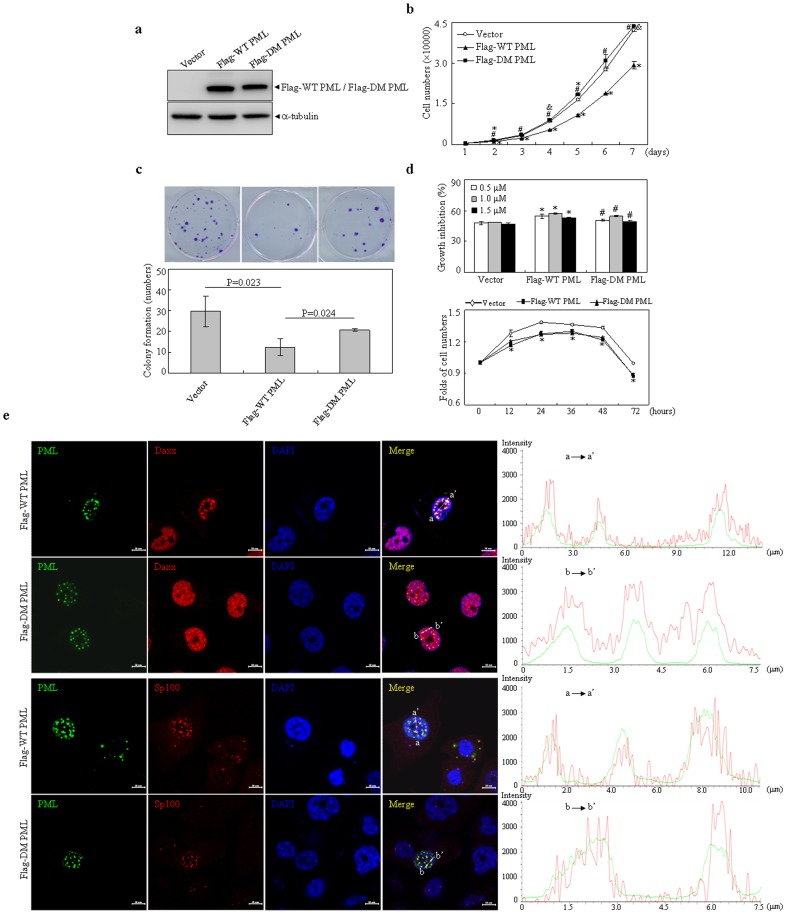
Effects of wild type and double mutant PML on growth and doxorubicin-induced cytotoxic activity of HEK293T cells. (a) HEK293T cells were stably transfected with indicated plasmids. The expressions of Flag tagged WT and DM PML proteins were detected with Flag antibody. (b) Indicated cells were respectively cultured for days as indicated and followed by CCK-8 assay. (c) Dense foci formation on a monolayer of indicated cells for 15 days was observed by light microscope (upper part) and foci numbers were counted. Data represents means with bar as S.D of three independent experiments (lower part). (d) Indicated cells were respectively treated with indicated concentrations of doxorubicin for 24 hours (upper part) or with 0.5 µM doxorubicin for hours as indicated (lower part), and followed by CCK-8 assay. Cell numbers were calculated as depicted in materials and methods. Cell growth was assessed by CCK-8 assay and relative folds against untreated cells were calculated. Data present means with bar as S.D of triplicate samples in an independent experiment. Symbols * and # respectively present p<0.05 compared with the cells expressing empty vector or Flag-WT PML. (e) PC3 cells were transfected with Flag tagged WT PML and DM PML expressing plasmids. After transfection for 24 hours, the cells were immunostainning with anti-Flag, Daxx or Sp100 antibodies. Representative images for colocalization of PML with Daxx or Sp100 were shown (scale bar  = 10 µM) and colocalization of Daxx or Sp100 within PML NBs were quantified by line scan analysis on left merged images. All experiments were repeated for three times and similar results were obtained.

To explore potential role of PML-interacting with LC3, Flag tagged WT and DM PML expressing plasmids were stably transfected into HEK293T cells, together with vector as control. As shown in Figure 8a, Flag tagged WT and DM PML demonstrated similar protein expression levels. Consistent with previous report [36], WT PML-expressing cells depicted a significant growth inhibition compared with that of vector expressing cells, as assessed by CCK-8 assay. Intriguingly, we found that DM PML expressing cells completely lost this growth inhibition effect (Figure 8b), suggesting that association of PML with LC3 may facilitate PML-conducted growth inhibition. Similar phenomena could also be found in colony formation assay when these transfected cells were cultured *in vitro* for 15 days (Figure 8c). Considering that PML has a pro-apoptotic property, we tested whether there is a difference between WT and DM PML expressing cells when treated with doxorubicin at different dosages. As assessed by CCK-8 assay, doxorubicin-induced cell growth inhibition was significantly increased in WT PML expressing cells, compared with empty vector expressing cells with corresponding treatments (upper part, Figure 8d), which was consistent with previous notion that PML presents pro-apoptotic function [37], [38]. DM PML expressing cells displayed similar growth inhibition effect of doxorubicin compared with that of WT PML expression although it depicted a little different response. Meanwhile, dynamic time-course analysis with doxorubicin (0.5 µM)-treated WT PML- and DM PML-expressing cells displayed a similar response to doxorubicin-induced cell growth inhibition, compared to that of vector-expressing cells (lower part, Figure 8d). Further, several PML nuclear body components including DAXX, Sp100, SUMO and UBC9 were surveyed for if any of these are missing from PML body, which may affect cell survival following doxorubicin treatment. The results showed that WT PML and DM PML presented a similar recruitment potential of these components in PML NBs, as determined by line scan analysis (Figure 8e). All together, these results suggested that interaction of PML with LC3 did not contribute to pro-apoptotic property of PML.

## Discussion

In this study, we report that PML interacts with LC3 in a cell type-independent manner as assessed by Co-IP assay and co-localization observation. Moreover, overexpressed PML significantly coprecipitates with endogenous and nulear LC3-II form. One previous report that LC3-II is found in nuclei of fresh rat hepatocytes by biochemical fractionation approach also supports the notion that nuclear LC3 is mainly LC3-II form [39]. Interaction of PML and LC3 persists in Atg5^−/−^ cells (Figure 5a), which have only LC3-I due to an autophagy defect that prevents processing of LC3-I to LC3-II. Co-IP data of Figure 1a and Figure 6b indicate a preferential interaction of PML with LC3-I. Therefore, we speculate that PML may interact with both LC3-I and -II forms. Actually, interaction of PML and LC3 is not dependent on autophagic activity (Figure 5). More importantly, a fraction of endogenous PML protein was found to be colocalized with LC3 protein, while this colocalization effect was further potentiated by IFNα induction (Figure 3), supporting that PML interacts with nuclear portion of LC3 protein under physiological conditions and this interaction effect can be potentiated by PML upregulation. Recently, several reports indicate that LC3 has a nucleo-cytoplasmic distribution and the mobility of EGFP-LC3 protein in nucleus and cytoplasm was substantially slower as analyzed by FRAP suggesting that LC3 shuttles between nucleus and cytoplasm areas [18]. LC3 contains a putative nuclear export signal but it has not a known nuclear localization signal (NLS). Therefore, its nuclear import could be mediated through an interaction with a protein containing an NLS. PML could possibly be this candidate protein. Further, we found that PML associates with LC3 during telophase and G1 phase but not in metaphase and anaphase, suggesting their interaction specifically occurred in some phases of cell cycle. This phenomenon and its potential significance deserved to be further addressed.

Substantial evidence suggests that LIR motif is essential for proteins interacting with LC3 or its related family proteins. Our results showed that two conserved LIR motifs and particular the second one in PML sequence are required for interaction of PML with LC3 (Figure 6–7), suggesting that PML may directly interact with LC3. We cannot exclude the possibility that other LC3-interacting proteins may contribute to this observed phenotype. In particularly, p62 (sequestosome 1) and phosphoinositide-binding protein ALFY, described as binding partners for LC3 directly or indirectly, have been recently found to colocalize in PML NBs when nuclear export is blocked by treatment of exportin-1 inhibitor leptomycin B [40]. Moreover, another possibility that other proteins localized in PML nuclear bodies may also contribute to this interacting effect needs to be elucidated in future. Identifying PML-interacting proteins may address this issue.

LIR-containing proteins include cargo receptors such as p62, the related neighbor of BRCA1 gene 1 (NBR1) and optineurin, members of the basal autophagy machinery, proteins associated with vehicles and their transport, Rab GTPase-activating proteins and specific signaling proteins that are degraded by selective autophagy [16], [32], [41], [42], [43]. Several proteins such as Rab7 effector protein FYCO1 and two Rab guanosine triphosphatase-activating proteins including OATL1 (TBC1D25) and TBC1D5 can specifically bind to Atg8 family proteins, although these proteins are not substrates for autophagy but they directly or indirectly modulate critical process of autophagy such as interaction with lipid phosphatidylinositol-3-phosphate [44], fusion between autophagosome and lysosomes [45] or mediation of autophagosome maturation [46]. Our previous study showed that PML-RARα expression increases constitutively autophagic activity, but it cannot interact with LC3 as assessed by localization of LC3 and PML-RARα [19]. Unlike PML-RARα in which the second potential LIR motif (aa 621–624) is missing due to gene translocation, PML over-expression can potentially increase sequestration of partial LC3 protein in PML NBs without enhancing autophagic activity, implying PML may control basic autophagy level by recruiting fraction of LC3 proteins within nuclear area. Therefore, experiments examining the role of PML in autophagy may elucidate their relationships. However, double mutant PML protein that lacks interaction capability with LC3 also did not affect the intracellular activity of autophagy as determined by detecting endogenous LC3 expression, which is similar to WT PML, suggesting that interaction of PML with LC3 may not directly participate in modulation of autophagy process. A previous study demonstrates that nuclear import-defective PML I targets early endosomes, and defective PML III, IV and V localize in late endosomes and lysosome [24], suggesting cytoplasmic portion of PML may associate with lysosome compartment, the critical organelle executing autophagy process.

Finally we elucidated potential role of PML-interacting with LC3. Our results showed that DM PML lost cell growth inhibition effect, which was conducted by WT PML, indicating that the interaction contributes to growth arrest function of PML. Relatively involved mechanism and whether this interaction could further influence other functions of PML (such as senescence and viral responses) deserved to be elucidated in future.

Collectively, our results propose that PML interacts with LC3 proteins and this interaction is dependent of LIR motifs and confers cell growth inhibition effect of PML. This work suggests that nuclear portion of LC3 may associate with PML to control cell growth for prevention and inhibition of cancer occurrence and development.

## References

[1] Melnick A, Licht JD (1999) Deconstructing a disease: RARalpha, its fusion partners, and their roles in the pathogenesis of acute promyelocytic leukemia. Blood 93:3167–3215. 10233871

[2] Kakizuka A, Miller WH Jr, Umesono K, Warrell RP Jr, Frankel SR, et al (1991) Chromosomal translocation t(15;17) in human acute promyelocytic leukemia fuses RAR alpha with a novel putative transcription factor, PML. Cell 66:663–674. 1652368 10.1016/0092-8674(91)90112-c

[3] Goddard AD, Borrow J, Freemont PS, Solomon E (1991) Characterization of a zinc finger gene disrupted by the t(15;17) in acute promyelocytic leukemia. Science 254:1371–1374. 1720570 10.1126/science.1720570

[4] Jensen K, Shiels C, Freemont PS (2001) PML protein isoforms and the RBCC/TRIM motif. Oncogene 20:7223–7233. 11704850 10.1038/sj.onc.1204765

[5] Batty EC, Jensen K, Freemont PS (2012) PML nuclear bodies and other TRIM-defined subcellular compartments. Adv Exp Med Biol 770:39–58. 23630999 10.1007/978-1-4614-5398-7_4

[6] Zhong S, Salomoni P, Pandolfi PP (2000) The transcriptional role of PML and the nuclear body. Nat Cell Biol 2:E85–90. 10806494 10.1038/35010583

[7] Salomoni P, Pandolfi PP (2002) The role of PML in tumor suppression. Cell 108:165–170. 11832207 10.1016/s0092-8674(02)00626-8

[8] Pearson M, Pelicci PG (2001) PML interaction with p53 and its role in apoptosis and replicative senescence. Oncogene 20:7250–7256. 11704853 10.1038/sj.onc.1204856

[9] Nakahara F, Weiss CN, Ito K (2014) The role of PML in hematopoietic and leukemic stem cell maintenance. Int J Hematol. 10.1007/s12185-014-1518-x PMC4096053 24488785

[10] Brown NJ, Ramalho M, Pedersen EW, Moravcsik E, Solomon E, et al (2009) PML nuclear bodies in the pathogenesis of acute promyelocytic leukemia: active players or innocent bystanders? Front Biosci (Landmark Ed) 14:1684–1707. 19273155 10.2741/3333

[11] Ruthardt M, Orleth A, Tomassoni L, Puccetti E, Riganelli D, et al (1998) The acute promyelocytic leukaemia specific PML and PLZF proteins localize to adjacent and functionally distinct nuclear bodies. Oncogene 16:1945–1953. 9591778 10.1038/sj.onc.1201722

[12] Nakatogawa H, Ichimura Y, Ohsumi Y (2007) Atg8, a ubiquitin-like protein required for autophagosome formation, mediates membrane tethering and hemifusion. Cell 130:165–178. 17632063 10.1016/j.cell.2007.05.021

[13] Mizushima N, Komatsu M (2011) Autophagy: renovation of cells and tissues. Cell 147:728–741. 22078875 10.1016/j.cell.2011.10.026

[14] Shpilka T, Weidberg H, Pietrokovski S, Elazar Z (2011) Atg8: an autophagy-related ubiquitin-like protein family. Genome Biol 12:226. 21867568 10.1186/gb-2011-12-7-226 PMC3218822

[15] Kabeya Y, Mizushima N, Yamamoto A, Oshitani-Okamoto S, Ohsumi Y, et al (2004) LC3, GABARAP and GATE16 localize to autophagosomal membrane depending on form-II formation. J Cell Sci 117:2805–2812. 15169837 10.1242/jcs.01131

[16] Birgisdottir AB, Lamark T, Johansen T (2013) The LIR motif - crucial for selective autophagy. J Cell Sci 126:3237–3247. 23908376 10.1242/jcs.126128

[17] Rogov V, Dotsch V, Johansen T, Kirkin V (2014) Interactions between Autophagy Receptors and Ubiquitin-like Proteins Form the Molecular Basis for Selective Autophagy. Mol Cell 53:167–178. 24462201 10.1016/j.molcel.2013.12.014

[18] Drake KR, Kang M, Kenworthy AK (2010) Nucleocytoplasmic distribution and dynamics of the autophagosome marker EGFP-LC3. PLoS One 5:e9806. 20352102 10.1371/journal.pone.0009806 PMC2843706

[19] Huang Y, Hou JK, Chen TT, Zhao XY, Yan ZW, et al (2011) PML-RARalpha enhances constitutive autophagic activity through inhibiting the Akt/mTOR pathway. Autophagy 7:1132–1144. 21673516 10.4161/auto.7.10.16636 PMC3210306

[20] Kuma A, Hatano M, Matsui M, Yamamoto A, Nakaya H, et al (2004) The role of autophagy during the early neonatal starvation period. Nature 432:1032–1036. 15525940 10.1038/nature03029

[21] Yan ZW, Hou JK, He W, Fan L, Huang Y (2013) Chloroquine enhances cobalt chloride-induced leukemic cell differentiation via the suppression of autophagy at the late phase. Biochem Biophys Res Commun 430:926–932. 23262180 10.1016/j.bbrc.2012.12.052

[22] Boichuk S, Hu L, Makielski K, Pandolfi PP, Gjoerup OV (2011) Functional connection between Rad51 and PML in homology-directed repair. PLoS One 6:e25814. 21998700 10.1371/journal.pone.0025814 PMC3187806

[23] Yu Y, Wang LS, Shen SM, Xia L, Zhang L, et al (2007) Subcellular proteome analysis of camptothecin analogue NSC606985-treated acute myeloid leukemic cells. J Proteome Res 6:3808–3818. 17655343 10.1021/pr0700100

[24] Jul-Larsen A, Grudic A, Bjerkvig R, Boe SO (2010) Subcellular distribution of nuclear import-defective isoforms of the promyelocytic leukemia protein. BMC Mol Biol 11:89. 21092142 10.1186/1471-2199-11-89 PMC2998510

[25] Stadler M, Chelbi-Alix MK, Koken MH, Venturini L, Lee C, et al (1995) Transcriptional induction of the PML growth suppressor gene by interferons is mediated through an ISRE and a GAS element. Oncogene 11:2565–2573. 8545113

[26] Regad T, Chelbi-Alix MK (2001) Role and fate of PML nuclear bodies in response to interferon and viral infections. Oncogene 20:7274–7286. 11704856 10.1038/sj.onc.1204854

[27] Palibrk V, Lang E, Lang A, Schink KO, Rowe AD, et al (2014) Promyelocytic leukemia bodies tether to early endosomes during mitosis. Cell Cycle 13:1749–1755. 24675887 10.4161/cc.28653 PMC4111721

[28] Levine B, Klionsky DJ (2004) Development by self-digestion: molecular mechanisms and biological functions of autophagy. Dev Cell 6:463–477. 15068787 10.1016/s1534-5807(04)00099-1

[29] Klionsky DJ (2007) Autophagy: from phenomenology to molecular understanding in less than a decade. Nat Rev Mol Cell Biol 8:931–937. 17712358 10.1038/nrm2245

[30] Nisole S, Maroui MA, Mascle XH, Aubry M, Chelbi-Alix MK (2013) Differential Roles of PML Isoforms. Front Oncol 3:125. 23734343 10.3389/fonc.2013.00125 PMC3660695

[31] Maroui MA, Pampin M, Chelbi-Alix MK (2011) Promyelocytic leukemia isoform IV confers resistance to encephalomyocarditis virus via the sequestration of 3D polymerase in nuclear bodies. J Virol 85:13164–13173. 21994459 10.1128/JVI.05808-11 PMC3233141

[32] Colecchia D, Strambi A, Sanzone S, Iavarone C, Rossi M, et al (2012) MAPK15/ERK8 stimulates autophagy by interacting with LC3 and GABARAP proteins. Autophagy 8:1724–1740. 22948227 10.4161/auto.21857 PMC3541284

[33] Liu L, Feng D, Chen G, Chen M, Zheng Q, et al (2012) Mitochondrial outer-membrane protein FUNDC1 mediates hypoxia-induced mitophagy in mammalian cells. Nat Cell Biol 14:177–185. 22267086 10.1038/ncb2422

[34] Sancho A, Duran J, Garcia-Espana A, Mauvezin C, Alemu EA, et al (2012) DOR/Tp53inp2 and Tp53inp1 constitute a metazoan gene family encoding dual regulators of autophagy and transcription. PLoS One 7:e34034. 22470510 10.1371/journal.pone.0034034 PMC3314686

[35] Seillier M, Peuget S, Gayet O, Gauthier C, N'Guessan P, et al (2012) TP53INP1, a tumor suppressor, interacts with LC3 and ATG8-family proteins through the LC3-interacting region (LIR) and promotes autophagy-dependent cell death. Cell Death Differ 19:1525–1535. 22421968 10.1038/cdd.2012.30 PMC3422476

[36] Kuo HY, Chen YC, Chang HY, Jeng JC, Lin EH, et al (2013) The PML isoform IV is a negative regulator of nuclear EGFR's transcriptional activity in lung cancer. Carcinogenesis 34:1708–1716. 23563092 10.1093/carcin/bgt109

[37] Bernardi R, Pandolfi PP (2003) Role of PML and the PML-nuclear body in the control of programmed cell death. Oncogene 22:9048–9057. 14663483 10.1038/sj.onc.1207106

[38] Guo A, Salomoni P, Luo J, Shih A, Zhong S, et al (2000) The function of PML in p53-dependent apoptosis. Nat Cell Biol 2:730–736. 11025664 10.1038/35036365

[39] Karim MR, Kanazawa T, Daigaku Y, Fujimura S, Miotto G, et al (2007) Cytosolic LC3 ratio as a sensitive index of macroautophagy in isolated rat hepatocytes and H4-II-E cells. Autophagy 3:553–560. 17617739 10.4161/auto.4615

[40] Clausen TH, Lamark T, Isakson P, Finley K, Larsen KB, et al (2010) p62/SQSTM1 and ALFY interact to facilitate the formation of p62 bodies/ALIS and their degradation by autophagy. Autophagy 6:330–344. 20168092 10.4161/auto.6.3.11226

[41] Johansen T, Lamark T (2011) Selective autophagy mediated by autophagic adapter proteins. Autophagy 7:279–296. 21189453 10.4161/auto.7.3.14487 PMC3060413

[42] Svenning S, Lamark T, Krause K, Johansen T (2011) Plant NBR1 is a selective autophagy substrate and a functional hybrid of the mammalian autophagic adapters NBR1 and p62/SQSTM1. Autophagy 7:993–1010. 21606687 10.4161/auto.7.9.16389 PMC3210314

[43] Alemu EA, Lamark T, Torgersen KM, Birgisdottir AB, Larsen KB, et al (2012) ATG8 family proteins act as scaffolds for assembly of the ULK complex: sequence requirements for LC3-interacting region (LIR) motifs. J Biol Chem 287:39275–39290. 23043107 10.1074/jbc.M112.378109 PMC3501051

[44] Pankiv S, Johansen T (2010) FYCO1: linking autophagosomes to microtubule plus end-directing molecular motors. Autophagy 6:550–552. 20364109 10.4161/auto.6.4.11670

[45] Itoh T, Kanno E, Uemura T, Waguri S, Fukuda M (2011) OATL1, a novel autophagosome-resident Rab33B-GAP, regulates autophagosomal maturation. J Cell Biol 192:839–853. 21383079 10.1083/jcb.201008107 PMC3051816

[46] Popovic D, Akutsu M, Novak I, Harper JW, Behrends C, et al (2012) Rab GTPase-activating proteins in autophagy: regulation of endocytic and autophagy pathways by direct binding to human ATG8 modifiers. Mol Cell Biol 32:1733–1744. 22354992 10.1128/MCB.06717-11 PMC3347240

